# Transient Ischaemic Attack (TIA) Knowledge in General Practice: a cross-sectional study of Western Adelaide general practitioners

**DOI:** 10.1186/1756-0500-5-278

**Published:** 2012-06-07

**Authors:** Elaine Stephanie Leung, Monica Anne Hamilton-Bruce, Cate Price, Simon A Koblar

**Affiliations:** 1 Stroke Research Programme, School of Medicine, University of Adelaide, Adelaide, South Australia, Australia; 2 Sturt Fleurieu General Practice Education and Training, Strathalbyn, South Australia, Australia; 3 Department of Neurology, The Queen Elizabeth Hospital, Woodville South, South Australia, Australia

**Keywords:** Transient ischaemic attack, General practitioners, Clinical guidelines, Medical education

## Abstract

**Background:**

With evidence to support early assessment and management of TIAs, the role of the general practitioner (GP) needs to be considered in developing a TIA service in Western Adelaide. We thus aimed to determine GP knowledge of TIA assessment and management and identify perceived barriers, in order to tailor subsequent GP education and engage primary care in the co-ordinated care of TIA patients.

**Findings:**

A self-administered questionnaire was mailed to all GPs (n = 202) in the Adelaide Western General Practice Network. Response frequencies were calculated for all variables, and associations examined by univariate analysis.

32 GPs responded. All respondents correctly identified early risk of stroke following a TIA. Difficulty accessing neurological expertise was identified as a barrier (40.6 %), as was a lack of GP knowledge (18.8 %). Areas for improvement included access to neurologists (36.7 %), relevant guidelines and education (43.3 %).

**Conclusions:**

Diagnosis of TIA is difficult and this study highlights the need for further education and practical guidelines for GPs. With this training, GPs could be better equipped to assess and manage TIAs effectively in the community in consultation with stroke physicians.

## **Findings**

### Background

The role of the general practitioner (GP) can be significant in the assessment and management of transient ischaemic attacks (TIAs). TIA patients may regard their symptoms with less urgency and present to primary care, and the diagnosis can be a difficult one. Increasing evidence supports early urgent assessment and management of TIAs to prevent subsequent stroke. An estimated 20 % of strokes are preceded by a TIA, with the risk of stroke following a TIA being between 10-20 % in the next 90 days [1], and half of these patients suffer a stroke within the first 48 hours [2]. A recent study of a 24-hour TIA clinic reported that 74 % of patients were discharged home after prompt assessment and treatment, potentially lowering costs [3]. Early assessment and initiation of treatment of TIAs has also been associated with an 80 % reduction of early subsequent stroke in another study by Rothwell et al [4]. However, the approach to care varies both nationally and internationally, with some advocating for admission and others suggesting ambulatory care.

There are currently no formal pathways for TIA care in Adelaide and it is unclear how GPs manage patients who present with suspected TIA in Australia. GPs in the Australian health system have a number of options including managing the patient themselves, referring onto an emergency department at either a public or private hospital, referring to a public neurology outpatient clinic with variable waiting times, referring to a private neurologist or to a TIA clinic is one exists in the region.

There have been some studies that have assessed the knowledge of GPs on stroke and TIA management. Middleton et al. assessed GPs’ knowledge of TIA/stroke risk factors and stroke prevention and management in New South Wales in 2003 [5]. They concluded that GPs required more purposeful and effective education. Their study, however, concentrated mostly on stroke and risk factors, and preceded recent knowledge about early stroke risk and stratification of TIAs. Other studies overseas similarly preceded current knowledge of early treatment and focused on the GPs ability to diagnose TIA[6-8]. The diagnosis of TIA is difficult, even amongst neurologists [9,10] and this study instead aimed to determine the knowledge of TIA assessment and current management amongst GPs, given the release of the NSF guidelines highlighting the need for urgent care. We also sought to identify perceived barriers to the assessment and management of TIAs locally that may influence future planning of services.

### Methods

Drawing on the postal questionnaire by Middleton et al, a questionnaire, comprising of 3 sections, was designed to evaluate the knowledge of GPs on the assessment and management of TIAs. Section one aimed to collect demographic data while the second section contained questions based on case scenarios. The case scenarios were written by the authors, two of whom are general practitioners and based on typical cases seen in general practice. The third section asked open questions enquiring about the perceived barriers to TIA assessment and current management in general practice. Participants were asked to comment on the barriers to assessment and management, and areas they considered required improvement.

All responses were read and general themes were extracted and coded into categories. With approval from the Royal Australian College of General Practitioners (RACGP) National Research and Evaluation Ethics Committee granted, the questionnaire was piloted on a group of 18 GP educators and supervisors to ensure that the questions were appropriate and would maximise response rates. Previous papers [11,12] have suggested methods to improve response rates, including a relevant topic, offering feedback, length of questionnaires, assurances of confidentiality, incentives, association with other stakeholders and personal contact. The pilot group addressed these and the initial questionnaire and cover letter were modified accordingly. Participants felt that a shorter questionnaire, ‘less exam like’, with assurances of feedback and confidentiality, paper based (rather than electronic) with a letter from the chief investigator (a GP registrar) rather than a well known academic would assist in encouraging GPs to participate.

With the assistance of the Adelaide Western General Practice Network (AWGPN), questionnaires were mailed to all 202 GPs on their database. The AWGPN covers an area of 205.4 square kilometres with the population of Western Adelaide reported as 212, 741 in 2006 [13,14]. The AWGPN funded the postage costs and stationery, and because of the *Privacy Act* 1988 were unable to disclose a list of GPs in the area. Subsequently the network’s administrative staff performed the mail out to GPs in the area. The questionnaire was accompanied by a covering letter explaining the purpose of the study, that the study was supported of the AWGPN Chief Executive Officer, a participant information sheet and consent form. A self-addressed pre-paid envelope was provided for participants to return the completed form and questionnaire to the investigators. An advertisement was included in the AWGPN newsletter in the month that the questionnaires were posted, inviting GPs to participate.

A follow-up reminder facsimile was sent to all GPs by the AWGPN 3 weeks following the mail-out. A random selection of GP names was then generated using a random number generator, and the investigator visited practices to raise awareness about the questionnaire amongst practice administrative staff. Practice managers were asked to remind GPs of the study and further copies of the questionnaire were provided. No financial or other incentive was offered with any invitation to GPs to participate in this study.

Questionnaires returned were de-identified and responses entered into a database. All questionnaires were included in the study although some had missing responses. Responses to the case scenarios were coded in true, false, unsure or missing categories, as the scenarios were not designed to be purely correct or incorrect responses. The coding was determined before data was collected. Statistical analysis of the data was undertaken using SPSS version 15.0, with frequencies for questionnaire responses calculated for all variables.

### Results

30 GPs responded to this questionnaire after the initial mailing and a further 2 responded after the follow up methods were employed. The response rate was 16 % from a total of 202 GPs invited to participate. A further two GPs returned questionnaires indicating they were not interested in participating (no reasons given) and three were addressed “return to sender”.

The demographic data collected is shown in Table 1.

**Table 1:** Demographics of respondents

	**Study data** (%)	**Australian data**[15] (n = 22868)
**Division of General Practice Worked**	(n = 30)	N/A
Adelaide Western General Practice Network	28	
Adelaide North East Division of General Practice	1	
Other	1	
**Member of the division**	(n = 29)	N/A
Yes	28	
No	1	
**Type of Practice**	(n = 30)	37 %
Solo	9 (30.0)	
Partnership	5 (16.7)	
Group	15 (50.0)	
Other	1 (3.3)	
**University**	(n = 31)	Australian graduates 68.6 %
University of Adelaide	22 (71.0)	Overseas 31.4 %
Flinders University	7 (22.6)	
Interstate	2 (6.4)	
Overseas	0	
**Year of graduation**	(n = 31)	NA
1940–1960	2 (6.5)	
1961–1980	13 (41.9)	
1981–2000	12 (38.7)	
2000-	4 (12.9)	
**Duration of GP experience (years)**	(n = 31)	NA
0–10	4 (12.9)	
11–20	11 (35.5)	
21–30	10 (32.2)	
31–40	4 (12.9)	
>41	2 (6.5)	
**Sessions worked per week**	(n = 31)	NA
>10	5 (16.1)	
9–10	13 (41.9)	
7–8	8 (25.8)	
5–6	2 (6.5)	
3–4	1 (3.2)	
1–2	1 (3.2)	
0	1 (3.2)	
**Worked in areas outside of general practice**	(n = 29)	NA
Yes	8 (27.6)	
No	21 (72.4)	
**Gender**	(n = 31)	62.0 %
Male	15 (48.4)	38.0 %
Female	16 (51.6)	
**Age (years old)**	(n = 31)	(<35) 9 %
20–30	2 (6.4)	(35–44 ) 25.1 %
31–40	2 (6.4)	(45–54 ) 32.4 %
41–50	10 (32.3)	(>54 ) 33.4 %
51–60	11 (35.5)	
61–70	4 (12.9)	
71+	2 (6.4)	
**Fellow of**	(n = 20)	NA
Royal Australian College of General Practitioners	16 (51.6)	
Australian College of Remote and Rural Medicine	1 (3.2)	
Other college	3 (10)	
**Stroke interest**	(n = 31)	NA
Yes	3 (9.7)	
No	28 (90.1)	
**Recent stroke/TIA education**	(n = 28)	NA
Yes	4 (14.3)	
No	24 (85.7)	

Most (n = 27) respondents have over 10 years of experience in general practice work, with 2 having had more than 41 years of experience. Most respondents (n = 18) were working more than 9 clinical sessions per week.

#### *Diagnosis of TIA*

The first case scenario asked questions about the diagnosis of TIA. The responses are presented in Table 2. The responses consistent with the current evidence are highlighted in bold. The current evidence for our assessment is included in the column headed as ‘Evidence’.

**Table 2:** Case scenario 1a

	**True**	**False**	**Unsure**	**Evidence**
	**n**	**n**	**n**	
1.She may have had a TIA	32	0	0	At the time of the study a TIA was defined as a sudden focal loss of neurologic function with complete recovery usually within 24 hours [16].The National Institutes of Health (NIH) committee on the Classification of Cerebrovascular Disease defined the time based definition of TIA. In 1965 the arbitrary 24-hour time limit definition was adopted, in a setting where there was limited imaging or treatments for stroke [17]. A tissue based definition has been adopted since, with TIA now being a transient episode of neurological dysfunction caused by focal brain, spinal cord, or retinal ischemia, without acute infarction [18].
2.She may have had a stroke	7	21	3	
3.A normal CT brain excludes a stroke	8	23	0	An early CT scan (within the first few hours) may be normal in ischaemic stroke. However, with experienced observers in up to 50 % of cases abnormalities can be seen on CT scan within 5 hours [19].
4.The differential diagnosis would include radiculopathy, cervical myelopathy or an intracranial pathology (e.g. tumour)	23	4	4	The diagnosis of TIA is clinical and can be challenging. The inter-observer diagnosis of TIAs even amongst neurologists has been reported to be poor [10]. The possible list of differential diagnoses can be extensive, ranging from significant neurological disorders to somatisation disorder.

Mrs JM, a 65 year old lady, presents with a history of tingling in her left arm and left leg whilst she was on holidays 2 weeks ago in Queensland. She smokes 8 cigarettes a day and is on Indapamide 2.5 mg daily for her hypertension. Her symptoms which lasted for about an hour resolved completely, and she thought that it was the hot weather that triggered it. Her BP today is 170/90. She is not a diabetic and her recent (total) cholesterol 7.9 mmol/L.

#### *Stratification of TIA risk*

The second part of the case evaluated knowledge about the risk of stroke following a TIA, wit h the results presented in Table 3.

**Table 3:** Case scenario 1b

	**True**	**False**	**Unsure**	**Evidence**
	**n**	**n**	**n**	
1.She would have been considered at low risk of stroke within 48 hrs of symptom onset	0	32	0	The risk of stroke following a TIA is significant, with a recent meta analysis reporting a 9.9 % risk of stroke after 2 days. [20]
2.Duration of symptoms does not contribute to risk	10	21	1	Factors that influence the risk of stroke include age, blood pressure, specific clinical features, presence of diabetes, duration of symptoms, aetiology of index event (e.g. atrial fibrillation), frequency of TIA symptoms, history of previous TIAs and smoking.
3.Limb weakness increases stroke risk	24	3	5	
4.BP contributes to risk of stroke in next 48 hrs	26	2	4	Johnston et al devised and validated a unified ABCD2 score to predict the risk of stroke after TIA at 2 days [21].

On further questioning you discover that she had some associated weakness but no speech symptoms. She denies any dizziness or headache.

#### *TIA investigations*

Another case scenario explored the possible investigations that could be arranged in primary care following a TIA. The results are presented in Table 4.

**Table 4:** Case scenario 3a

	**True**	**False**	**Unsure**	**Evidence**
	**n**	**n**	**n**	
1.A repeat CT scan in 7 days should be performed	6	11	15	Whilst diagnosis of a TIA is a clinical one, the use of imaging enables clinicians to confirm ischaemia, exclude haemorrhage or any other pathology mimicking a stroke. A CT scan after 8–10 days however, is less sensitive to haemorrhage and an MRI may be the more appropriate investigation [22].
2.Carotid duplex need not be done as symptoms were not in the carotid territory	5	25	2	As ‘best clinical practice’ the National Stroke Foundation [23] recommends that patients with carotid territory symptoms who would be candidates for surgery have a carotid duplex ultrasound. However, the reliability in determining the correct vascular territory clinically is only moderate in neurologists [24]. Bloods should be obtained routinely in all patients for a full blood picture, electrolytes, renal function, fasting lipids, erythrocyte sedimentation rate and/or C-reactive protein and glucose. An ECG should be performed in all patients, with attention to the presence of atrial fibrilliation (AF).
3.Bloods should be taken for FBE, ESR, BGL, lipids,UEC	31	1	0	
4.ECG not needed as PR is regular	2	28	2	

Mrs FH is a 58 year old lady who is discharged from the hospital Emergency Department yesterday following a TIA with symptoms of vertigo and ataxia, which have completely resolved. She presents to you for follow up having had a normal CT brain in the Emergency Department but no other investigations.

#### *TIA management*

The following case scenario explored the options of assessment and possible referral in a general practice setting. The results are presented in Table 5.

**Table 5:** Case scenario 2

	**True**	**False**	**Unsure**	**Evidence**
	**n**	**n**	**n**	
1.As symptoms have resolved there is no urgency in the assessment and management	2	30	0	Although the symptoms have resolved the risk of stroke remains significant. The ABCD^2^ score for this patient is 7 and would place him at high risk of a subsequent stroke. A score of 6 or 7 was found to have an 8.1 % risk of subsequent stroke in the following 48 hours [21].
2.Management in GP setting with CT before starting aspirin	20	9	3	The patient’s score is considered high risk, with the NSF recommending that a CT brain be performed within 24 hours [23].
				Whilst the use of aspirin after a CT is recommended, a study of 9000 patients randomised to aspirin without CT found no significant excess haemorrhages, even in those who had an initial haemorrhagic stroke [25]. However, in practice CT brain is performed prior to commencing aspirin.
				Admission to an ASU would allow comprehensive monitoring and early access to treatment including thrombolysis if appropriate if this patient were to develop a subsequent stroke but the evidence remains unclear as to the best model of care.
3.Refer patient to neurology outpatients	7	20	4	
4. Best practice would be to have him admitted to an Acute Stroke Unit (ASU).	16	7	9	

Mr DM is a 61 year old man who presents with a suspected TIA. His symptoms included weakness in his right arm yesterday, which resolved after 2 hours. He has a history of diabetes but has been managed on diet alone. He is an ex- smoker and his father had a stroke at 70 years. He has a history of hypertension for which he is on Perindopril 10 mg daily. His BP today is 150/68 and there are no significant neurological findings on examination.

The final questions related to the instigation of treatment for the secondary prevention of stroke, with the results presented in Table 6.

**Table 6:** Case scenario 3b

With regards to treatment the following statements are true or false.				
	**True**	**False**	**Unsure**	**Evidence**
	**n**	**n**	**n**	
1.Aspirin or aspirin/dipyridamole should be started	29	1	1	Studies have demonstrated that antiplatelet treatment significantly reduces the risk of stroke [26], with the combination of aspirin and dipyridamole shown to be more effective than aspirin alone [27].
2.Clopidogrel is 1^st^ line	5	22	4	Trials continue to assess the benefits of clopidogrel in stroke prevention with some studies suggesting that it is more effective than aspirin alone. However, the MATCH trial compared Clopidogrel and clopidogrel with aspirin and found no significant difference [28]. The NSF suggests that clopidogrel should be considered for those intolerant of aspirin or if aspirin is contraindicated [23].
3.Referral for carotid endarterectomy(CEA) if duplex reveal ipsilateral carotid stenosis of 70-99 %	22	2	7	Carotid endarterectomy has been found to reduce the risk of disabling stroke or death for patients with stenosis exceeding ECST-measured 70 % or NASCET-measured 50 %, in surgically-fit patients operated on by surgeons with low complication rates (less than 6 %) [29].
4.ECG reveals AF and warfarin should be started	31	0	1	A Cochrane review in 2004 concluded that anticoagulation can reduce the risk of stroke in patients with non- rheumatic atrial fibrillation (AF) [30]
5.A lipid lowering agent (statin) should be started only if her blood test reveal hypercholesterolaemia	9	20	3	Whilst earlier trials suggested increased rates of intracerebral haemorrhage and concerns were raised about liver toxicity, recent studies have demonstrated a modest decrease in stroke risk with statin therapy [31].
6.Anti-hypertensive should be commenced regardless of BP	15	12	5	Evidence suggests that patients should receive BP lowering treatment after a TIA unless contraindicated by symptomatic hypotension [32].

#### *Perceived barriers to assessment and management from a primary care perspective*

Open questions then sought to explore the perceived barriers to the assessment and management of TIAs in general practice. Responses were analysed and themes extracted.

Difficulty in accessing neurological expertise or acute stroke units (ASU) was identified as a barrier by 13 respondents, whilst accessing investigations for the assessment of TIAs was considered a barrier by 7. The lack of knowledge both by GPs (n = 6) and the public (n = 7) was also identified as a barrier to TIA assessment and management. The lack of time in general practice consultations was identified by 5 of the 32 GPs in the survey as a barrier to effectively manage potential TIA cases.

In response to questioning about the areas for improvements, participants addressed the barriers identified earlier. Improved access to neurologists and/or ASUs (n = 11) and better access to investigations (n = 2) were suggested. The establishment of relevant guidelines and specific education for GPs (n = 13) and public education (n = 6) were also considered as areas for improvement.

Participants were asked about their preferences for attending educational workshops. Participants indicated a conference venue as the most preferred venue (n = 22), followed by GP division offices (n = 17), own clinic (n = 6) and RACGP offices (n = 1) as the least preferred.

### Discussions

The case scenarios suggested that respondents were less confident in selecting specific treatments in TIA, with 15/32 answering correctly about anti-hypertensive treatment and slightly more correct with respect to managing hyperlipidaemia (20/32). However all correctly identified the early risk of stroke following a TIA, and nearly all answered correctly on the appropriate blood tests to order in a TIA case. The diagnosis of TIA is recognised as difficult, and this study highlights that whilst knowledge on the assessment and risks of TIA is present, there is a need for further education and practical guidelines for GPs to improve knowledge with respect to specific management and pathways of care. The National Stroke Foundation Audit observed the decline in public hospital based TIA clinics in 2007 [33] and with the best model of TIA care yet to be established, the current system may be failing to address the needs of the community for efficient TIA assessment and management. Potentially low risk TIAs could be managed appropriately in general practice, and thus contribute to ease the burden on the public hospital system. With training, GPs could be better equipped to assess and manage low risk TIAs effectively in the community.

Given the current medical workforce climate, it was not surprising that difficulty in accessing neurological specialist opinion and hospital acute stroke units, in particular, were identified as a barrier. However, access to private radiological investigations, were not as notably identified. Miller et al report that GPs order a CT at a rate of 1 per 100 encounters and of these 2 % are for cerebral ischaemia [34]. The current Medical Benefits Scheme (MBS) does not allow GPs to request MRI scans.

Whilst respondents identified the lack of public knowledge about TIA symptoms, they were also aware of their own knowledge deficits. The National Stroke Foundation developed the *Clinical Guidelines for Acute Stroke Management* in 2007 in line with NHMRC standards, and included recommendations for TIA management. However, 6 respondents considered that there was a lack of knowledge and relevant guidelines to assist their practice. Similarly 7 respondents answered that there was a lack of knowledge amongst their patients with respect to the symptoms of TIA, thus resulting in late presentations for medical care. State-based Stroke Associations and the NSF provide information for consumers on stroke and TIA. However, there is limited evidence on the current public knowledge of stroke/TIA and the effects that an educational intervention will have. There is however some evidence that information and education for patients who have suffered a stroke will improve patient and carer knowledge of stroke, aspects of patient satisfaction, and reduce patient depression scores [35].

The long-term treatment goals of secondary prevention constitute the daily work of GPs. However, with continued workforce shortages in primary care, GPs face time pressures in providing comprehensive care to the community. Multidisciplinary care plans have been introduced by government initiatives and this may provide incentives to appropriate management of TIA [36]. Whilst the work of GPs routinely includes educating patients, with limited consultation time, education of the public needs to be addressed at a broader level. With regular liaison between community care and tertiary level hospitals, GPs can and should be able to recognise TIA early, as well as contribute to assessment and management.

#### *Limitations*

The response rate from the questionnaire limits this study, as the small sample of GPs may not be representative and open to bias. Previous studies have acknowledged the difficulty in engaging GPs to participate in postal surveys and have suggested a number of techniques [37]. The questionnaire and cover letter were piloted first and amendments made to optimise the response rate. This study was undertaken by a GP Academic Registrar and no financial or other incentive was offered to invited participants which may have improved the response rate. The most common reason that medical practitioners decline involvement in surveys is time [38]. Methods to improve response rates to surveys have included an advance phone prompt from medical personnel or a small gift with the survey [39]. The Canadian National Physician Survey attempted to improve their response rates by implementing a number of strategies including a monetary incentive but were unsuccessful [40]. Others have suggested that there is no optimal response rate and whilst a high response rate is more likely to be representative of the sample, a low response rate may be valid if non-response effects are tested [41,42]. The follow-up methods employed in this study included a personal visit to random practices, as a ‘personal’ approach has been considered as important [43]. However, the contact details of invited GPs were not accessible to the investigators under the *Privacy Act* and the use of personally addressed letters and specific follow up of non-responders was not performed. The support of the Division was considered to be important, with previous studies reporting that appropriate stakeholders involvement would assist in improving response rates and thus use of a commercial list of GPs was not used [44].

GPs in the AWGPN constitute 12 % of the South Australian GP workforce. The AWGPN registers all GPs working in the area as members by default, but 4/32 participants replied that they did not consider themselves members of this division. Most respondents reported that they worked in group practices (n = 15) whilst 9 (30 %) respondents indicated that they were solo practitioners, compared to 37 % nationally [15]. 31.4 % of GPs nationally are overseas trained but none of the respondents in this study were trained overseas. The majority were female (n = 16) whilst nationally 62 % of GPs are male and most were over 41 years age, which is comparable to national data. Those invited to participate in the study were registered as GPs working within the area of AWGPN as supplied by their database, however, with the current workforce status there has been a fluctuation of GPs in and out of practices with 4/32 respondents indicating that they did not currently work in the AWGPN. The demographic details of the participants were mostly consistent with available Australian data, with the exception of location of training. Those who reported to be members of a division and/or fellows of a professional college were more likely to participate. Surprisingly, despite the limited time GPs give to participate in surveys, the majority of participants unexpectedly worked more than 8 sessions per week. The implementation of guidelines successfully depends in part on their applicability to a local region [45] and thus this study aimed to determine the knowledge in the Western Division of General Practice in Adelaide. However, there is no data available on the characteristics of GPs in this area and comparison was thus limited to available Australian data.

The low response rate may suggest a disinterest in the topic, which is cause for concern as TIA assessment and management is in the domain of general practice and the risk of subsequent morbidity and mortality significant. Previous studies have shown that non-responders are more likely to be older, more experienced, solo practitioners, more stressed and less well qualified than responders[44] with the one of reasons for not participating other than time was that the topic was thought to not be relevant. If non-responders are less qualified, their knowledge in TIA assessment and management may also be less than that of the study participants.

Whilst there may have been value in having a control group to compare the results to, for example neurologists, we would expect different answers as the approach to an acute neurological episode in general practice has its own challenges and barriers, which is what the study aimed to evaluate.

The questionnaire itself has its limitations. Whilst the use of a pre- and post- test questionnaire may have provided additional information on the retention of knowledge, this questionnaire was not designed to necessarily contain “right” and “wrong answers”. In order to maximise the response rate, and the authors wished to avoid an exam style approach to “testing” GPs. Similarly with a pre and post questionnaire GPs with good knowledge and interest may be more likely to participate and so presenting a biased sample. The authors consider instead that designing an educational intervention based on the questionnaire results, and then testing GPs after the education session may be more useful. Again though the questionnaire would have its limitations as it only suggests what GPs might do in an ideal clinical setting, which may be quite different to what occurs in real practice.

Since the study was conducted further research has been published on the assessment and management of TIA, which may have an impact on whether the responses are viewed as correct or incorrect. In particular the definition of TIA is now tissue based [18], and it may be worthwhile surveying GPs again to determine their awareness of this new definition and the subsequent changes to their clinical practice.

Within these limitations, this study nonetheless is suggestive of a need to improve knowledge amongst GPs, in particular the management pathways for TIA. A number of barriers to TIA care, including difficulties in accessing services, were also identified. Together with the low response rate, it seems that specific education to GPs to highlight the relevance and importance of this topic along with a review of the accessibility of services locally needs to be addressed so that we might in future contribute more effectively in caring for patients with TIAs in primary care.

### **Availability of supporting data**

The data set supporting the results of this article are available in the Stroke Research Programme repository, http://www.adelaide.edu.au/srp/.

### **Ethical approval**

The Royal Australian College of General Practitioners National Research and Evaluation Ethics Committee granted approval for this study.

## **Abbreviations**

TIA, transient ischaemic attack; GP, General practitioner; NSF, National Stroke Foundation; ASU, Acute Stroke Unit; RACGP, Royal Australian College of General Practitioners; AWGPN, Adelaide Western General Practice Network; MBS, Medicare Benefits Scheme; MRI, Magnetic Resonance Imaging; NHMRC, National Health and Medical Research Council.

## **Competing interests**

Funding: ESL was supported by Sturt Fleurieu General Practice Education and Training and the University of Adelaide in a GP Registrar Academic Post.

MHB, CP and SAK declare that they have no competing interests.

## **Authors’ contributions**

EL, AHB and SK contributed to the conception and design. EL carried out the collection and assembly of data, and the data analysis and interpretation. All authors contributed to manuscript writing and review, and approved the final manuscript.
